# An Approach to the Automated Characterization of Out-of-Plane and In-Plane Local Defect Resonances

**DOI:** 10.3390/ma16083084

**Published:** 2023-04-13

**Authors:** Paweł Zdziebko, Mateusz Krzemiński, Maciej Okoń, Gabriela Loi, Francesco Aymerich, Łukasz Pieczonka, Andrzej Klepka

**Affiliations:** 1 Department of Robotics and Mechatronics, AGH University of Krakow, Al. Mickiewicza 30, 30-059 Krakow, Poland; zdziebko@agh.edu.pl (P.Z.); mkrzemin@agh.edu.pl (M.K.); lukasz.pieczonka@agh.edu.pl (Ł.P.); 2 Department of Mechanical, Chemical and Materials Engineering, University of Cagliari, Piazza d’Armi, 09123 Cagliari, Italy; gabriela.loi@unica.it (G.L.); francesco.aymerich@unica.it (F.A.)

**Keywords:** local defect resonance, damage detection, structural dynamics, non-destructive testing, laser vibrometry, structural health monitoring

## Abstract

The paper presents an approach to efficiently detect local defect resonances (LDRs) in solids with localized defects. The 3D scanning laser Doppler vibrometry (3D SLDV) technique is applied to acquire vibration responses on the surface of a test sample due to a broadband vibration excitation applied by a piezoceramic transducer and modal shaker. Based on the response signals and known excitation, the frequency characteristics for individual response points are determined. The proposed algorithm then processes these characteristics to extract both out-of-plane and in-plane LDRs. Identification is based on calculating the ratio between local vibration levels and the mean vibration level of the structure as a background. The proposed procedure is verified on simulated data obtained from finite element (FE) simulations and validated experimentally for an equivalent test scenario. The obtained results confirmed the effectiveness of the method in identifying in-plane and out-of-plane LDRs for both numerical and experimental data. The results of this study are important for damage detection techniques utilizing LDRs to enhance the efficiency of detection.

## 1. Introduction

The increasing use of advanced materials in modern engineering structures demands new inspection strategies offering reliable and accurate data. In recent years, a wide range of non-destructive testing (NDT) methods and structural health monitoring (SHM) techniques have been developed for assessing the structural integrity of engineering materials [1,2,3]. Among them, methods based on the analysis of non-linear vibration/acoustic phenomena, such as higher and subharmonics generation and elastic waves modulation, have been gaining special attention [2,3,4,5,6,7,8]. This is mainly due to their better sensitivity to small damage severities than their linear counterparts. In this context, a novel non-invasive procedure that uses sonic or ultrasonic frequency excitation tuned to the local dynamic properties of defect to activate a resonant response was recently proposed by Solodov et al. [9]. The principle behind this is that the presence of embedded defects results in a local loss of stiffness that gives rise to characteristic resonant frequencies of the defect itself, known as Local Defect Resonances (LDRs). As for the classical modal testing approach, the match between excitation and LDR frequency corresponds to the maximum wave–defect interaction. The energy delivered by the impinging wave is selectively trapped within the damaged area, leading to a significant increase in the defect response amplitude. This increase is strongly localized in the defect area, providing an excellent contrast between the damage and the intact specimen. Solodov et al. further applied the concepts of LDR to a Flat Bottom Hole (FBH) [10]. Experimental results were validated through numerical modeling, and an analytical formulation was proposed to determine the LDR frequencies of defects such as FBH, delaminations in composite materials, and laminar defects in rolled sheet metals. The effects of local defects on the non-linear ultrasonic response were also investigated in the literature, revealing that the defect excited at its LDR frequency exhibited a transition to a non-linear regime. Consequently, the input energy was efficiently converted into non-linear frequencies components, such as sub- and higher harmonics inside the damaged region [11,12]. LDR frequencies have also been used to enhance the thermal response of a damaged structure [13]. Solodov et al. [14] developed an analytical solution for different planar defect shapes, which was validated through a series of experimental tests. In the experiments, a wide-bans chirp signal was used to excite the specimens, and the laser vibrometer was used for response measurements. Laser Doppler vibrometry has been used by other researchers to extract LDR frequencies of aluminum and PMMA plates with FBHs, and delaminations in glass-fiber reinforced polymer (GFRP) and carbon-fiber reinforced polymer (CFRP) [15,16]. Moreover, it has been demonstrated that LDR behavior does not limit itself to out-of-plane direction but can be extended towards in-plane characteristics [17,18]. Experiments on different types of defects, i.e., FBHs, surface cracks and BVIDs (Barely Visible Impact Damage), showed a clear in-plane LDR at an elevated frequency range due to the high in-plane bending stiffness.

Even though many papers deal with the LDR frequencies for imaging damage, only a few attempts were made to develop a robust algorithm to identify the LDR frequencies among the system’s natural frequencies, making the procedure cumbersome and time-consuming, starting from the analytical formulation proposed in [14], which can be applied only for a few classes of idealized defects and only when the geometry and position of the fault are known. More recently, in [19,20], the authors proposed an approach based on the bicoherence analysis to obtain the LDR of FBH in an aluminum plate and of delamination in a GFRP composite plate.

The current study proposes a novel algorithm for the efficient detection of LDRs. The main goal of the algorithm is to automate the process of identifying LDR frequencies, which is a particularly tedious task for structures with a considerable number of resonances. It is designed to exhibit unique features, including:

- Possibility of investigating structures with multiple defects;
- Detection of multiple frequencies (and their mode shapes) for the same defect, which is needed for in-plane LDRs extraction associated with higher-order modes;
- Possibility of examining structures without a priori knowledge of defects’ locations or limits regarding their size.

The above-listed features make the proposed approach novel with respect to the existing approaches, such as the algorithm proposed in [12], which is based on a similar index to that presented in this paper. However, the processing procedure of the proposed index is modified to improve the algorithm’s features.

In this paper, we present the work carried out to develop, validate and verify the effectiveness of the method of searching the LDRs (both out-of-plane and in-plane) in the broad spectrum of vibration modes. Firstly, we describe the algorithm in Section 2. The test sample, experimental setup, and finite element models are presented in Section 3. Damage detection and localization results are presented in Section 4. Finally, the paper is concluded in Section 5.

## 2. Materials and Methods

**Figure 1:**
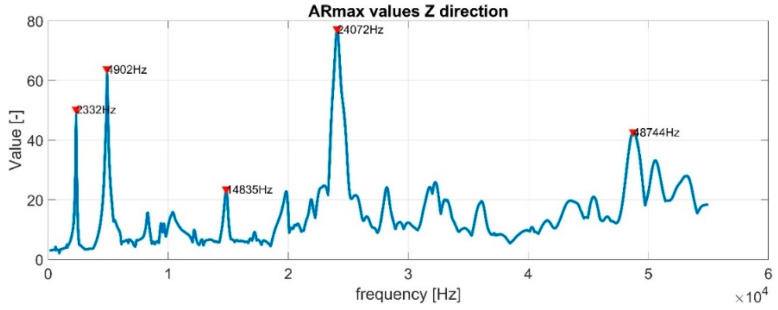
Example of the ARmax characteristic.

As a result of the local loss of stiffness, the LDRs are specific for the selected area of a structure. Hence, vibrations in the corresponding frequencies are increased in damaged regions, and a negligibly small background response is observed simultaneously. It indicates that for the local modes, the vibration amplitudes in the areas of damage are significantly higher than the mean value of the amplitudes of the whole structure. Based on this observation, we introduce the Average Ratio (*AR*):

(1) $$AR_{i,f}=\frac{A_{i,f}}{E\left[A_{f}\right]},$$

where $A_{i,f}$ is the velocity amplitude of vibration of the single point *i* in the single frequency *f*, and $E\left[A_{f}\right]$ is the mean value of amplitudes of all points in the single frequency. Having frequency response functions (FRF) for all the points of a structure, we can create the *M* × *N* matrix of absolute FRF values, where *M* is the number of points and *N* is the number of frequencies. This allows for the calculation of the *AR* matrix. Then, selecting the maximum *AR* value in each column gives the *ARmax* characteristic (see Figure 1).

**Figure 2:**
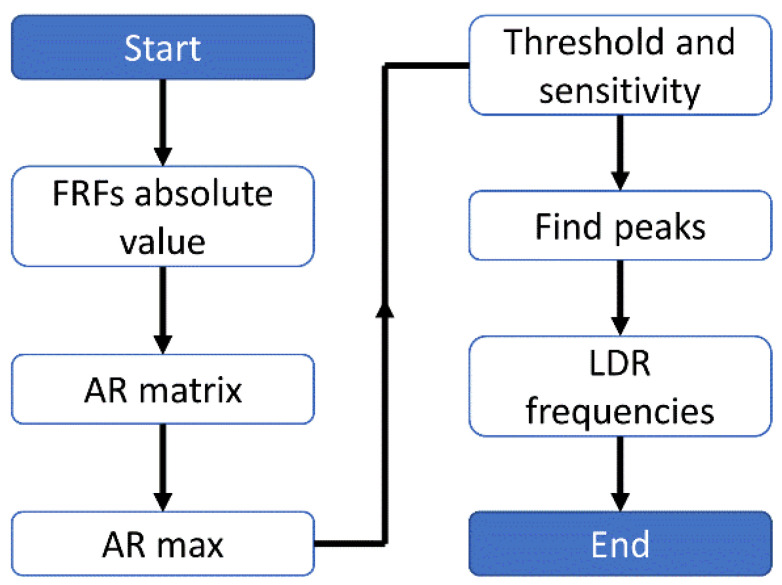
The flowchart of the automated LDR extraction algorithm.

The *ARmax* characteristic contains information about the relative differences between points with the highest amplitudes and mean values for a whole structure. The higher the value is, the more distinct LDR is detected. Two parameters determine the threshold for LDR frequency selection. The first one is the sum of the mean and standard deviation of prominence to define the minimum peak prominence. The second one is the standard deviation of *ARmax* values, respectively, for the whole characteristic to define minimum peak height. The algorithm’s sensitivity is controlled by the maximum number of peaks to be detected. Usually, it is set to 9–24. The concept and main steps of the calculations are schematically summarized in Figure 2.

To sum up, the algorithm works as follows: the FRF computed for measured or simulated data is the input for the algorithm. Firstly, the absolute value of FRF is computed. Next, the *AR* matrix is calculated according to Equation (1) and the *ARmax* index is calculated as introduced above. Then, the peaks of the *ARmax* are found, taking into account threshold and sensitivity presets. Frequencies corresponding to those peaks are interpreted as LDR frequencies.

## 3. Examination Setup

### 3.1. Test Sample with FBHs

**Figure 3:**
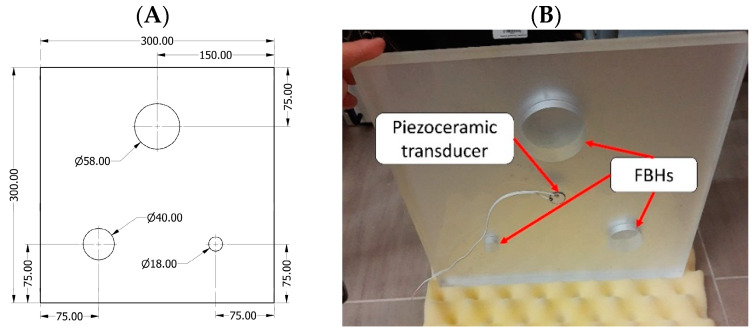
PMMA test sample with FBHs: nominal dimensions in [mm] (**A**) and the sample prepared for experiments (**B**).

The test sample made of poly(methyl methacrylate) (PMMA) was manufactured for the experimental testing of the algorithm’s operation. The dimensions of the plate are as follows: 300 × 300 × 18 mm. Damages of FBH-type were introduced in the plate to represent three sizes of deep defects with nominal diameters of 58, 40, and 18 mm. The designed depth of all FBHs was 17 mm, which corresponds to the 1 mm thickness of the residual material in the damaged area. The test specimen’s dimensions are presented in Figure 3A. Please note that the sample is presented from the intact-side view, while in Figure 3B the manufactured sample is presented from the damaged-side perspective.

A preliminary study revealed that non-negligible differences were observed between the simulated and experimentally measured natural frequencies, which was not expected for a relatively simple structure under consideration. Therefore, we decided to verify the geometrical dimensions of the sample with respect to their desired nominal values in order to tune the numerical model accordingly. The dimensions of FBHs of the test sample are summarized in Table 1.

It has been confirmed that the differences observed between the designed and measured dimensions of FHBs were the reason for the inconsistencies between the preliminary results of the numerical calculations and the experiments. The following formula allows for the prediction of the LDR frequencies for FBHs, and it was used to determine the influence of the variation of FBHs’ dimensions on LDR frequencies:

(2) $$f_{LDR}=\frac{6.4t}{a^{2}}\sqrt{\frac{E}{12\rho \;\left(1-v^{2}\right)}}$$

where t is the defect’s residual material thickness, a is the diameter of the FBH, E is the Young’s Modulus, ρ is the density, and v is the Poisson ration of the material.

**Figure 4:**
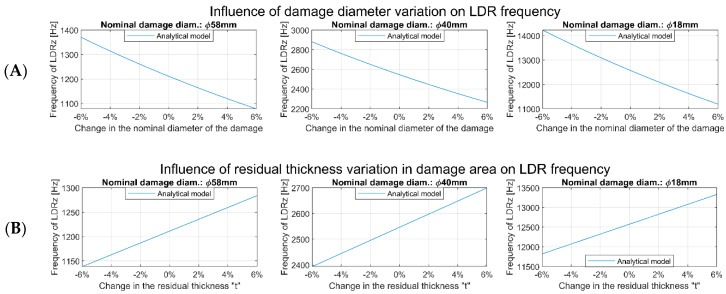
Influence of damage diameter (**A**) and residual thickness (**B**) variation on theoretical LDR frequencies calculations made for the design values of FBH dimensions.

The data shown in Figure 4 prove that a slight change in the residual thickness or the diameter of the damage can significantly shift the LDR frequency.

### 3.2. Experimental Modal Analysis

**Figure 5:**
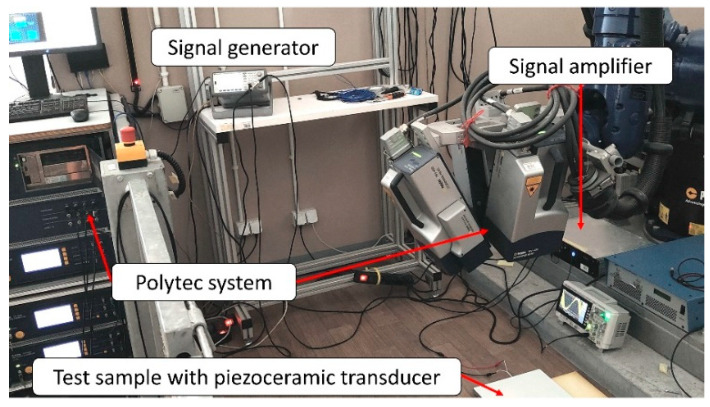
Measurement system used in experiments—configuration with piezoceramic transducer.

In experiments, the sample was freely suspended to avoid boundaries’ nonlinearities. Preliminary studies have shown that a model shaker is required to excite the largest FBH’s normal modes effectively. At the same time, the piezoceramic transducer was needed to excite higher-frequency modes of LDRs. A frequency sweep signal was used to excite the sample in the following frequency ranges: 0.5–3 kHz using a modal shaker and 0.5–20 kHz in the case of using a piezoceramic transducer. The excitation amplitudes were set as 0.2 V and 8 V for the shaker and piezoceramic transducer, respectively. An external signal generator generated the signal, and next the power amplifier amplified it ten times and passed it on to the piezoceramic transducer. In the case of using a modal shaker, the generated signal was amplified using a built-in amplifier. In both cases, the test sample’s response was measured using 3D scanning laser Doppler vibrometry (3D SLDV). The sample surface was mapped with 362 equally spaced measurement grid points. The *Polytec PSV400 3D* laser vibrometer was used for non-contact measurements of vibration responses. The sampling frequency and single point measurement duration were 5 kHz and 1.6 s in the shaker measurement and 125 kHz and 2.048 s in the piezoceramic transducer measurement. For both acquisitions, we used three averages per point. The Frequency Response Functions (FRFs) were calculated from the experimental input and output data using the *Polytec PSV* software v9.0. The experimental arrangement for measurements is presented graphically in Figure 5.

### 3.3. Numerical Models

Numerical simulations can be used to predict the dynamic properties of a structure. In the context of this work, a computational modal analysis and FRF synthesis were used to generate input data for the identification algorithm. Two numerical models employing the Finite Element Method (FEM) to simulate the test specimen with FBHs were formulated. The first model was based on 3D 8-node brick Finite Elements (FEs) and is assumed to accurately reflect the real structure’s response. The second proposed model, based on 2D 4-node shell FEs, is supposed to allow for a rough determination of LDRs in a shorter computing time. Both models were formulated using the *Altair HyperMesh* v2020 software and computed with the *MSC.Nastran* v2020 solver.

**Figure 6:**
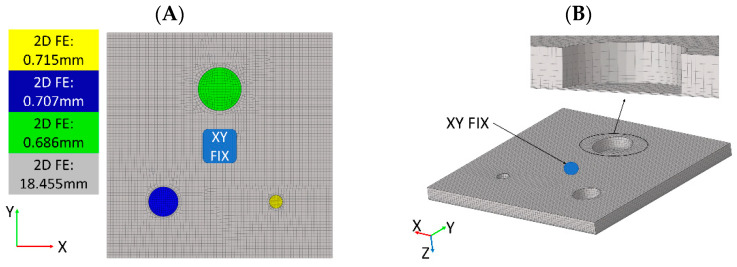
Formulated numerical models based on 2D FEs (**A**) and 3D FEs (**B**).

Figure 6 presents the FE mesh used in the 2D and 3D numerical models. The geometric dimensions of the model correspond to the measured ones. The thickness of shell 2D FEs corresponds to the thickness of the material in a given area of the sample. The thicknesses of shell FEs used in the 2D model are described in the legend in Figure 6A. The influence of the piezoceramic transducer was modeled as constraints in X and Y directions on the transducer’s montage side. The division of elements in the XY plane is the same in both models.

A mesh convergence analysis was carried out, comparing the first LDR frequency of the damage. According to the outcomes of this analysis, the recommended number of FEs per FBH diameter was determined as 16. Three FEs were modeled on the thickness of the residual material in the damaged area in the case of the 3D model and eleven FEs in the intact area of the plate. The material parameters adopted in the simulations are summarized in Table 2.

**Figure 7:**
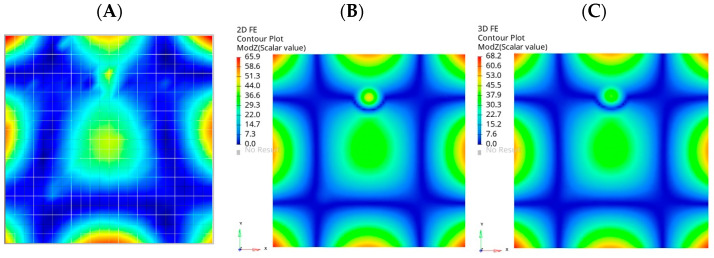
One of the global normal modes selected for validation: experiment (**A**), 2D FE model (**B**), 3D FE model (**C**).

A comparison of selected normal models was carried out to check the correctness of the material model parameters. The numerical results of the natural frequencies of the test sample were compared with the measured ones. One of the analyzed normal modes is presented in Figure 7—hereinafter referred to as global mode ‘B’.

Analogous comparisons were made for two other global normal modes, called ‘A’ and ‘C’. The results of the validation are presented in Table 3.

The obtained results confirm a good agreement of the numerical model formulated with 3D FEs with the experimental results. The maximum error was observed for the normal mode marked as ‘B’, which was less than 5% between the model and experiment. This error is more significant for the model made of 2D FEs, and amounts to a maximum of 7.6% for the ‘C’ normal mode. The model made of 3D FEs can be considered to be more accurate. It should be noted that the thickness of the test sample in numerical models was assumed to be constant, but in reality it most likely varied over the sample, which may be the reason for the discrepancy between numerical results and experimental ones in global normal modes.

## 4. Results and Discussion

### 4.1. Out-of-Plane LDRs

**Figure 8:**
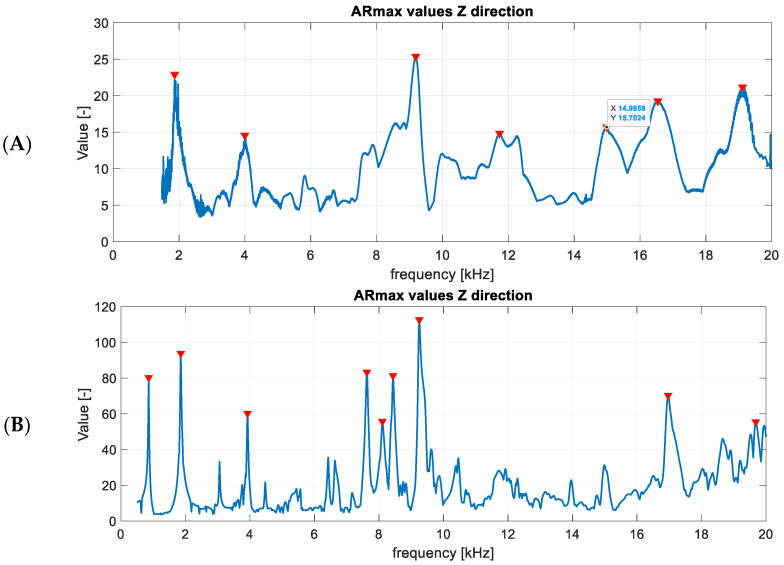
Computed ARmax characteristics and LDR frequencies selected by the algorithm for experimental data (**A**) and the numerical results (**B**).

Four datasets for the detection of out-of-plane LDRs were examined. The results were derived from experiments with a piezoceramic transducer, a modal shaker (described in Section 3.2) and the numerical results (2D and 3D FE models defined in Section 3.3). The determined *ARmax* characteristics for datasets were computed and analyzed, and finally LDR frequencies were found by the proposed algorithm. Exemplary results of the *ARmax* index for experimental data and the 3D numerical model are presented in Figure 8. Similar results were computed for the 2D numerical model, but are not presented here to limit the manuscript’s size. The maximum number of peaks to be detected in the algorithm was limited to nine. The LDR frequencies determined by the algorithm are marked with red triangles.

**Figure 9:**
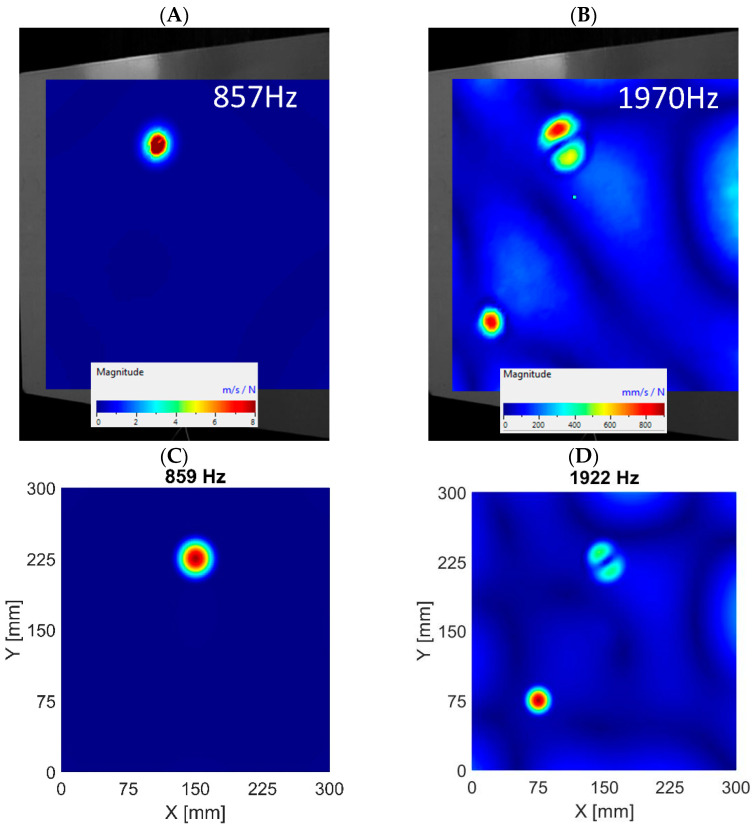
Visualization of out-of-plane vibrations—FBH Ø58 mm. The first LDR in experimental data (**A**) and numerical results based on the 3D FE model (**C**). The second LDR in experimental data (**B**) and numerical results based on the 3D FE model (**D**).

**Figure 10:**
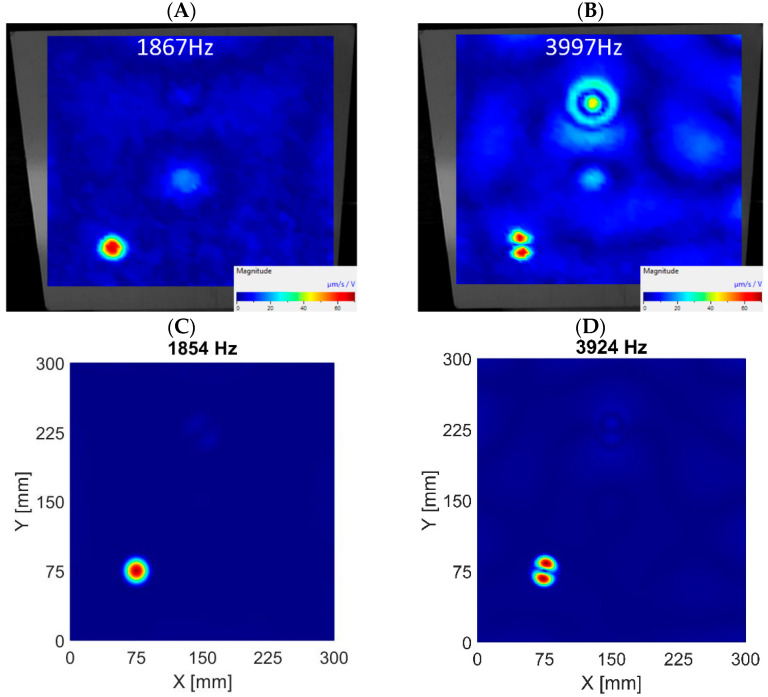
Visualization of out-of-plane vibrations—FBH Ø40 mm. The first LDR in experimental data (**A**) and numerical results based on the 3D FE model (**C**). The second LDR in experimental data (**B**) and numerical results based on the 3D FE model (**D**).

**Figure 11:**
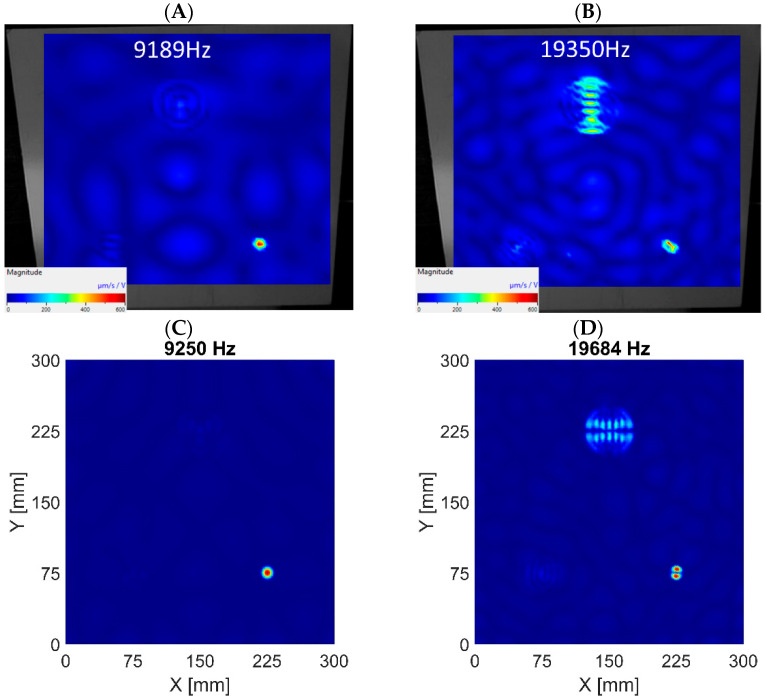
Visualization of out-of-plane vibrations—FBH Ø18 mm. The first LDR in experimental data (**A**) and numerical results based on the 3D FE model (**C**). The second LDR in experimental data (**B**) and numerical results based on the 3D FE model (**D**).

When comparing numerical and experimental results, it can be noticed that the damping in the actual structure is higher than in the numerical model, as the ratio of amplitudes shown in the *ARmax* characteristic is lower. The higher frequencies in the real system are damped more. The numerical models consider a 0.3% modal damping, which is the same for all modes. Normal mode shapes corresponding to the first and second LDRs were determined by the algorithm and are visualized in Figure 9, Figure 10 and Figure 11 for subsequent datasets. The results are presented for experimental data and numerical ones using the 3D FE model. Similar results were obtained for the 2D FE model.

The visualizations of LDRs of the FBH Ø58 mm allow for determining its location and shape. The algorithm identified similar frequencies to LDRs for numerical and experimental data. The first out-of-plane LDR turned out to occur below 1 kHz, so it was necessary to use a modal shaker instead of a piezoceramic transducer in the experimental setup. Piezo-stack was used to excite the structure at higher frequencies, but it was ineffective in the low-frequency range. It resulted in heavily noised signals, so using the detection algorithm on low-quality data did not make sense. For this reason, the tests for the low-frequency range were repeated using a modal shaker. It allowed for correct excitation of the first mode of out-of-plane LDR for the largest FBH. Unfortunately, the excitation of the second out-of-plane mode of LDR of FBH Ø58 mm was accompanied by the first out-of-plane mode of LDR of FBH Ø40 mm. As a result, the average vibration level was high, and thus the *ARmax* index was low. The same observations were noted in experimental and numerical results (both 2D and 3D models)—see Figure 9B,D. This caused a false negative indication for the second out-of-plane LDR mode of FBH Ø58 mm. It must also be noted that the algorithm also found higher LDRs, but the presentation is limited to the first two ones (first and second out-of-plane modes).

In the FBH Ø40 mm case, the algorithm correctly identified the first two modes of out-of-plane LDRs. The discrepancies between the experimental data and the results of the 3D model are minor, as presented in Figure 10. However, it should be added that these conclusions apply only to the model based on 3D FEs. The algorithm working on the numerical results from the 2D FEs model did not correctly indicate the second out-of-plane mode of LDR of FBH Ø40 mm (false negative indication). On the other hand, the first out-of-plane mode was correctly extracted.

The FBH Ø18 mm is the only defect for which the algorithm’s indications are only true positive. It applies to all datasets, i.e., experimental data and 2D and 3D FE models. The visualizations of first and second out-of-plane LDRs of FBH Ø18 mm extracted by the algorithm for various datasets are depicted in Figure 11.

The summary of detected modes for three FBHs is presented in Table 4. The LDR frequencies determined by the algorithm were compared for different datasets to assess the accuracy of the numerical models. The summary is given in Table 4, and is limited to the first and second detected LDR frequencies for given defects and presents relative errors for the two proposed FE models (based on 2D and 3D FEs). It can be observed that the results obtained from numerical models correspond very closely with the experimental results, which prove the successful validation of the numerical models. As expected, when focusing on the first out-of-plane mode of the LDR, the 3D model turned out to be more accurate—the maximum error was observed for FBH Ø40 mm, and it amounted to 0.7% compared to the experimental data.

The maximal error in the 2D model was observed for the FBH Ø18 mm, which amounted to 2.9% compared to the experimental data, but it was still an acceptable result in most applications. Similarly, when comparing the second out-of-plane mode of LDRs, the 3D FE model proved slightly more accurate. The maximum error was observed for FBH Ø18 mm, but the difference was not as evident as for the first out-of-plane mode of LDRs, i.e., 1.73% and 2.18% for 3D and 2D FE models, respectively. It must also be noted that for any datasets, the algorithm found the second out-of-plane mode of FBH Ø58 mm. This problem was addressed earlier in the text. Unfortunately, the second out-of-plane mode of FBH Ø40 mm was also not recognized by the algorithm working with 2D FE model results.

It can therefore be summed up that 3D modeling gives more realistic results. In the case of 2D modeling, the results showed a more significant maximum divergence compared to the experiment than the results of the 3D FEs model. The algorithm working on the experimental data and the results from the 3D model correctly indicated the second out-of-plane mode of LDR (FBH Ø40 mm), which was not shown in the 2D data. On the other hand, the 2D model deserves attention, as it is much faster to formulate and is characterized by a three times lower computing time than for the 3D model. It can be successfully employed for a rough determination of the first out-of-plane LDR modes. Moreover, the 2D FE model allows the easy manipulation of the material’s thickness, saving a lot of time in the model-tuning process. The simulations were performed on a workstation with an AMD Ryzen^®^ 9 5950X 16-core processor.

### 4.2. Damage Size Assessment

**Figure 12:**
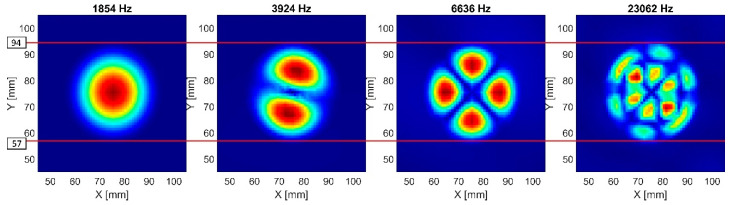
Comparison of the diameter of FBH Ø40 mm based on different LDR modes.

The higher order out-of-plane LDR modes allowed for a more accurate identification of the FBH damage size. Figure 12 presents a visualization of the detected out-of-plane modes of FBH Ø40 mm using the discussed algorithm. The view was limited only to the damaged area. The algorithm’s sensitivity was increased to 24 peaks across the entire frequency spectrum. It allowed for the identification of higher out-of-plane LDRs of the FBH Ø40 mm damage. Horizontal lines were drawn to allow the quantification of the damage size based on the shape of the highest noted mode (23,062 Hz). The determined size of the damage was Ø37 mm. This indication was therefore underestimated in relation to the nominal damage diameter by 3 mm. Nevertheless, the lower modes underestimated the damage diameter to a much greater extent. The first out-of-plane mode, especially, underestimated the damage diameter by about 15 mm, which resulted in a damage size of Ø25 mm.

### 4.3. In-Plane LDRs

The proposed algorithm successfully detects out-of-plane modes of LDRs. Nonetheless, another examination was performed to check the possibility of identifying the in-plane LDRs using the presented algorithm. In this case, the *ARmax* characteristic was computed based on the velocity amplitude of vibrations in the XY plane instead of the velocity Z component. In the case of experimental data, visualization was provided with the Y (vertical) component of vibration velocities. The algorithm’s sensitivity was set to 24 because in-plane LDRs are usually related to higher mode shapes.

**Figure 13:**
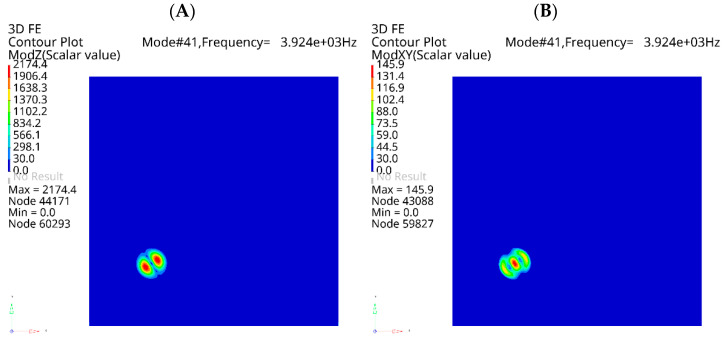
One of the mode shapes of FBH Ø40 mm LDR: out-of-plane component (**A**) and in-plane component (**B**).

Although the analysis concerned in-plane modes, most detected resonance frequencies corresponded to out-of-plane modes of LDRs. Further investigation showed that out-of-plane modes are, in fact, always accompanied by increased vibrations in the in-plane directions, as presented in Figure 13, where for the same eigenvalue (3925 Hz) the out-of-plane (Figure 13A) and in-plane (Figure 13B) vibration components are depicted.

**Figure 14:**
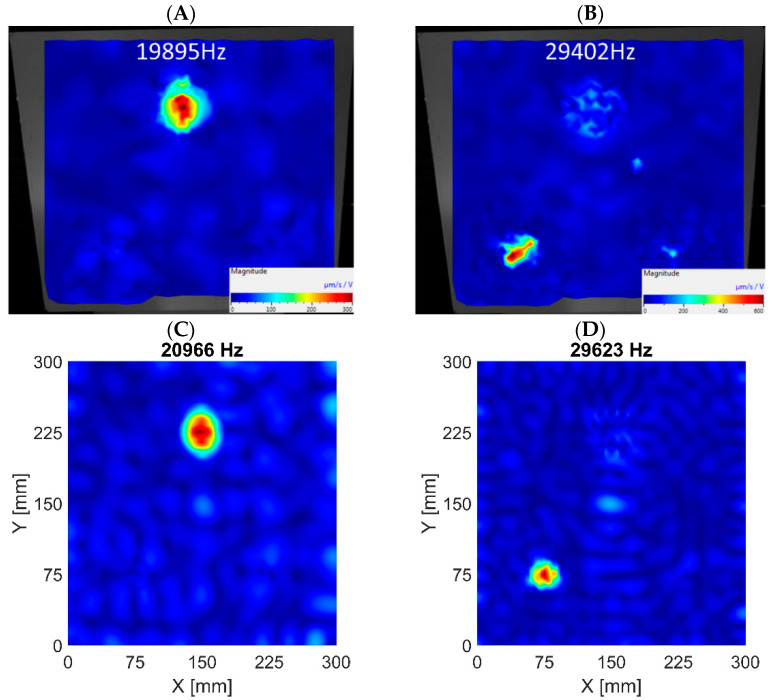
‘Clear’ in-plane LDRs of FBH Ø58 mm based on experimental data (**A**) and 3D FE model results (**C**); ‘Clear’ in-plane LDRs of FBH Ø40 mm based on experimental data (**B**) and 3D FE model results (**D**).

The assessment of the normal modes determined by the algorithm showed that all indications referred to the FBHs introduced into the plate. None of the indications was a false-positive, proving the validity of the proposed approach. As mentioned earlier, most indications referred to the out-of-plane modes, for which significant in-plane vibrations were also observed. For this reason, these indications cannot be considered incorrect even though they do not correspond to ‘clear’ in-plane modes. In the determined results, two modes could be recognized as ‘clear’ in-plane modes. Those modes refer to the Ø58 mm and Ø40 mm FBHs, and are visualized in Figure 14. No ‘clear’ in-plane LDRs of FBH Ø18 mm were found, which is likely to be out of range of the analysis. The summary of detected in-plane LDRs is presented in Table 5.

In the case of using the algorithm to identify in-plane LDR modes, the developed approach also proved to be very effective. It can be noticed that the 3D numerical model allows the representation of in-plane modes of FBHs more accurately than the model based on 2D FEs. The errors reported for the 2D FE model (more than 13%) questioned the correctness of in-plane LDR frequency representation for this modeling technique. The observed differences with the experimental results were more significant than for the out-of-plane modes for both FE models. It was associated with a worse representation of real structures by numerical models in the higher frequency range.

## 5. Conclusions

This paper presents a method for determining the frequencies of local defect resonances. The algorithm was based on the observation that the vibration amplitudes were significantly higher for the local defect’s mode shape than the mean value of the whole structure. Based on that observation, the *ARmax* was computed as a ratio between the maximum amplitude of vibrations for a given frequency and the mean vibrations level. The algorithm found the *ARmax* index’s peaks and identified them as LDR frequencies.

To sum up, the most significant achievements reported in this paper are as follows:

- A novel algorithm for the automatic extraction of LDRs was developed. The algorithm was successfully implemented to identify out-of-plane and in-plane modes of LDRs. Various datasets, including experimental and numerical data of a test specimen with FBH of different diameters, were examined;
- The algorithm allowed the determination of higher-order LDR modes, which usually better represented FBH shape and size. Finding higher-order LDRs was also essential to correctly extract in-plane modes. This feature was a significant improvement compared to the current state-of-the-art methods;
- Two numerical models based on the FEM were developed. One employed 3D FEs, and the other was based on 2D FEs. The algorithm worked successfully with the results provided by both models;
- The indicated LDRs proved that, in most cases, the 2D model was sufficient for determining FBH-type out-of-plane LDRs. The recorded error between corresponding LDRs in experiments and results of the 2D FE model was less than 3%. On the other hand, a false negative indication was noted for this dataset. The results provided by the 3D FE model were better fitted with an actual structure, which corresponded with the relative error of less than 2% in out-of-plane modes;
- The 3D FE numerical model allowed the capturing of in-plane modes of FBHs more accurately than the 2D FE model. The errors of in-plane LDRs called into question the applicability of the 2D modeling technique to represent in-plane modes.

It should be emphasized that the presented work is the basis for further research. Future works will concern the use of the developed algorithm for LDRs detection for other types of damage, such as delaminations in composites or fatigue cracks.

## Figures and Tables

**Table 1:** Design and measured dimensions of the test sample.

Dimension Type	Nominal Value	Identified Values
Thickness of plate	18 mm	18.455 mm
FBH “Ø58 mm” diameter	58 mm	57.646 mm
FBH “Ø58 mm” residual thickness	1 mm	0.686 mm
FBH “Ø40 mm” diameter	40 mm	39.64 mm
FBH “Ø40 mm” residual thickness	1 mm	0.707 mm
FBH “Ø18 mm” diameter	18 mm	17.566 mm
FBH “Ø18 mm” residual thickness	1 mm	0.715 mm

**Table 2:** Material properties of the material used in numerical models.

Parameter	Value
Young’s modulus	4919 MPa
Poisson ratio	0.4
Density	1.204 g/cm^3^

**Table 3:** Natural frequencies of the test sample.

		Model with 2D FEs		Model with 3D FEs	
Global Mode	Experiment	Simulation	Error	Simulation	Error
‘A’	478 Hz	476 Hz	0.4%	480 Hz	0.4%
‘B’	1207 Hz	1121 Hz	7.1%	1151 Hz	4.6%
‘C’	2360 Hz	2181 Hz	7.6%	2296 Hz	2.7%

**Table 4:** Comparison of out-of-plane LDRs frequencies detected by the algorithm for numerical models and experiments.

			The Model with 2D FEs		The Model with 3D FEs	
	Defect Type	Experiment	Simulation	Error	Simulation	Error
First LDR (out-of-plane)	FBH Ø58 mm	857 Hz	855 Hz	0.23%	859 Hz	0.23%
	FBH Ø40 mm	1867 Hz	1859 Hz	0.43%	1854 Hz	0.70%
	FBH Ø18 mm	9189 Hz	9455 Hz	2.90%	9250 Hz	0.67%
Second LDR (out-of-plane)	FBH Ø58 mm	Not found	Not found	---	Not found	---
	FBH Ø40 mm	3997 Hz	Not found	---	3924 Hz	1.83%
	FBH Ø18 mm	19,350 Hz	19,771 Hz	2.18%	19,684 Hz	1.73%

**Table 5:** Comparison of in-plane LDRs detected by the algorithm for numerical models and experiments.

		The Model with 2D FEs		The Model with 3D FEs	
Defect Type	Experiment	Simulation	Error	Simulation	Error
FBH Ø58 mm	19,895 Hz	23,164 Hz	16.43 %	20,966 Hz	5.38%
FBH Ø40 mm	29,402 Hz	33,443 Hz	13.74 %	29,623 Hz	0.75%
FBH Ø18 mm	Not found	Not found	---	Not found	---

## Data Availability

Data available upon request.
